# Oral and dental health in head and neck cancer survivors

**DOI:** 10.1186/s41199-016-0015-8

**Published:** 2016-10-19

**Authors:** Firoozeh Samim, Joel B. Epstein, Zachary S. Zumsteg, Allen S. Ho, Andrei Barasch

**Affiliations:** 1 Department of Oral Medicine Oral Pathology, University of British Columbia, Vancouver, BC Canada; 2 Samuel Oschin Comprehensive Cancer Institute, Cedars-Sinai Medical Center, Los Angeles, CA USA; 3 Radiation Oncology, Samuel Oschin Comprehensive Cancer Institute, Cedars-Sinai Medical Center, Los Angeles, CA USA; 4 Department of Surgery, Samuel Oschin Comprehensive Cancer Institute, Cedars-Sinai Medical Center, Los Angeles, CA USA; 5 Department of Medicine, Weill Cornell Medical College, New York, NY USA

**Keywords:** Cancer treatment protocol, Cancer pre-treatment protocol, Oral hygiene, Oral complication management, Head and neck cancer survivors

## Abstract

Therapeutic improvements and epidemiologic changes in head and neck cancer (HNC) over the last three decades have led to increased numbers of survivors, resulting in greater need for continuing management of oral and dental health in this population. Generally, the HNC patient oral health needs are complex, requiring multidisciplinary collaboration among oncologists and dental professionals with special knowledge and training in the field of oral oncology. In this review, we focus on the impact of cancer treatment on oral health, and the oral care protocols recommended prior to, during and after cancer therapy. The management of oral complications such as mucositis, pain, infection, salivary function, taste and dental needs are briefly reviewed. Other complications and their management, including osteonecrosis of the jaw and recurrent/new primary malignancies are also described. This review offers clinical protocols and information for medical providers to assist in understanding oral complications and their management in HNC patients and survivors, and their oral and dental health care needs. Oral and dental care is impacted by the patient’s initial oral and dental status, as well as the specific cancer location, type, and its treatment; thus, close communication between the dental professional and the oncology team is required for appropriate therapy.

## Background

More than 600,000 cases of HNC are diagnosed each year worldwide. With evolving etiologies and advances in treatment, HNC survival has improved in recent decades and the population of HNC survivors continues to grow [1]. This increasing number of survivors raises additional challenges, particularly in the management of those patients who have complex medical, oral/dental, and psychosocial needs. Management of the oral complications in this population typically requires multidisciplinary collaboration among different professionals and healthcare providers, including head and neck surgeons, medical oncologists, radiation oncologists, and dental professionals with special knowledge and training in the field of oral oncology.

HNC may present with oral manifestations, which necessitates recognition and appropriate referral/treatment [2, 3]. Generally, therapy for malignant diseases affects the mouth both directly through cytotoxicity, and indirectly through the effects on immune function or other systemic side effects. Common oral complications include pain, mucositis, salivary gland dysfunction, taste loss/change, infections, dysphagia, fibrosis, soft tissue and/or bone necrosis, exacerbation of dental and periodontal diseases, and recurrent or secondary malignancy [4]. The incidence and severity of oral complications are affected by cancer treatment modality(s) used, anatomic location and stage of the cancer, extent of oral or dental diseases prior to treatment, comorbidities and genetic risk, oral hygiene and nutrition [5]. To effectively prevent as well as treat oral complications, dental professionals play important roles prior to, during and following active cancer treatment [6].

For HNC survivors, it is generally recognized that trained/experienced dental professionals serve as integral components of the multidisciplinary oncology team. These dental professionals can play a role in prevention of oral complications through patient education (improve oral hygiene, maintain nutrition, reduce alcohol and tobacco use), treatment of dental disease, prophylactic strategies and treatment of oral complications, and early detection of oral malignancy in high-risk patients [7]. The related referral pathways are through dental and medical specialists with oncology experience [8]. This review focuses on the role of dental health care providers, and protocols of oral care in HNC patients.

## Head and neck cancer treatment and its impact on the oral cavity

All treatment modalities for HNC produce oral complications, including surgery (e.g. mutilation and physiologic changes), radiation therapy (e.g. mucositis, dysphagia, hyposalivation, osteoradionecrosis), and neoadjuvant, adjuvant and/or concurrent chemotherapy (e.g. mucositis, taste changes, immune suppression) [9]. Additionally, newer targeted therapies may also result in oral mucosal complications [10, 11]. These medications include epidermal growth factor inhibitors, which cause erythematous mucosal reactions; tyrosine kinase inhibitors and mammalian target of rapamycin (mTOR) inhibitors, both of which may cause isolated aphthous-like lesions; and emerging immunotherapies, which may induce lichenoid reactions [12]. Given the commonality of oral complications of therapy, pre-treatment oral conditions may affect therapeutic options selected for some patients [2]. Additionally, the long-term oral sequelae of these treatments require oral and dental follow-up and fastidious long-term oral care.

Oncologists need to recognize the importance of pre-treatment dental care, and use available resources to effectively address oral needs [13]. For improved outcomes, it is imperative that a thorough oral assessment, implementation of basic oral care protocols, management of pre-existing dental conditions, and prevention and management of emerging oral complications be completed before HNC treatment. This is best accomplished by integrated teams with oncology-trained/experienced dental providers who provide timely dental treatment and preventive protocols that do not disrupt cancer therapy.

The following sections provide a summary of evidence-based guidelines related to oral/dental management in HNC patients, with the goal of improving health outcomes and enhancing long-term quality of life.

## Before HNC therapy

It is important to be aware that some practicing dentists may have limited experience in care of the oncology patient, and that dental professionals with oncology experience may be required to identify and manage oral conditions and diseases in HNC patients [14]. According to the Multinational Association of Supportive Care in Cancer/International Society of Oral Oncology (MASCC/ISOO) guidelines, thorough basic oral care is recommended to all cancer patients for control of bacterial flora and reduction of inflammatory and/or infectious complications [15, 16]. Oral hygiene recommendations are shown in Table 1.

**Table 1:** Oral care prior to and post cancer treatment in head and neck cancer survivors

Pre – Cancer Treatment	o Pre-treatment assessment 2–3 weeks prior to cancer therapy o Comprehensive head and neck, oral mucosa, dental and periodontal examination o Radiographs to assess dental and periodontal status o Baseline jaw range of motion (interincisal opening), baseline resting and stimulated saliva o Advanced caries, advanced periodontal disease: definitive treatment may require surgery with goal of 1–2 weeks of healing time o Periodontal debridement maintenance; oral hygiene instruction o Custom fluoride carriers, custom oral positioning devices
During Cancer Treatment	o Individual treatment as cancer type and planned treatment indications o Oral hygiene reinforced o Small carious lesions may be treated with fluoride and/or sealants; daily fluoride applications o Symptom management: Pain: topical analgesic and anesthetic agents; systemic analgesics; dry mouth: hydration, oral rinses and coating agents; lip management o Mucositis reduction: Patient education: o Regular brushing, flossing; prosthesis cleaning o Bland oral rinses, water based/wax or lanolin lip lubricant o Fluoridated toothpaste; or home fluoride trays daily in high risk patients o Soft toothbrushes; Electric or ultrasonic brushes for tolerated patients o Super-soft brush for severe mucositis or foam brush with chlorhexidine if brushing not possible o Dietary instruction; nutritional guidance, tobacco and alcohol avoidance
Post – Cancer Treatment	o Monitoring, prevention and management of oral complications (mucositis, dry mouth, mucosal pain, taste change, infection, dental demineralization, dental caries, periodontal disease, soft tissue/osteonecrosis etc.) o Checking for cancer recurrence or secondary primary cancer o Dental caries prevention, periodontal maintenance o Determine frequency of dental hygiene follow-up interval based on level of hyposalivation, demineralization/caries rate and patient’s oral hygiene post-radiotherapy; patients with dry mouth, may require hygiene and recall every 3–4 months o Patient education o Fluoridated toothpaste; in high risk patients home fluoride trays daily o Good oral hygiene, soft toothbrushes or electric or ultrasonic brushes, flossing o Maintain lubrication of mouth and lips o Encourage non-cariogenic diet and cessation of tobacco & alcohol

HNC patients should receive a comprehensive oral assessment prior to any cancer treatment, as soon as possible after diagnosis, and ideally, within 2 to 3 weeks prior to beginning cytotoxic therapy, in order to allow time for healing if surgical dental procedures are indicated [17–19]. The assessment should include full periodontal examination, radiographic examination, salivary gland functional assessment, and jaw range of motion measurement. Other treatments, such as non-surgical dental needs and stabilizing dental conditions can be completed as needed, or postponed until after cancer therapy, if deemed elective [20, 21].

Individual HNC patients should be managed according to cancer therapy-specific pre-treatment dental protocols [22]. Absence of symptomatic and occult oral disease is the desired goal, and elimination of acute and chronic infections that may require future surgical care is indicated prior to cancer therapy. Oral tissues within the high dose radiation fields must receive particular attention, as post-radiation surgery in this region may be risky. Custom oral devices (positioning/opening devices, midline blocks or anti-scatter trays) may be prescribed to minimize radiation exposure to oral structures unaffected by cancer and to assist in tissue positioning in order to support radiation exposure to movable tissues on a repeated basis. Compromises in timing and extent of dental treatment may be necessary, particularly when tumor burden is excessive, but generally, the healthier the mouth, the better the odds for good outcomes [21, 22].

## During HNC treatment

It is essential that oncologists and other physicians involved in cancer care communicate with trained dental professionals in complex and unique cancer cases [23] so that necessary treatment is provided in timely fashion, unnecessary treatment is avoided, and preventive protocols are instituted. Even though cancer patients may be inclined to discontinue oral hygiene due to discomfort, the avoidance of basic hygiene results in increased microbial loads, gingival/oral inflammation and risk of infections. Thus, maintenance of oral hygiene should be encouraged. If oral hygiene is compromised during cancer treatment, the daily use of aqueous chlorhexidine 0.12 % solution can control the overall microbial load, including fungal and yeast overgrowth [24]. Devices built for prophylactic purposes (midline blocks, custom trays, etc.), must be used according to protocol.

Oral pain, predominantly due to mucositis, is one of the major symptoms in radiation therapy with or without chemotherapy. The biology of mucositis has been described and is leading to study of interventions based on the pathogenesis of the condition [25]. For pain management due to mucositis, MASCC/ISOO recommends topical analgesic/anesthetic agents (e.g., lidocaine, benzocaine, diphenhydramine), or in severe cases, patient-controlled analgesia (parenteral morphine sulphate). Study of potential prevention of mucositis is ongoing, with recent suggestion for use of low-energy laser (LLLT), which may significantly reduce the severity and duration of mucositis [2, 14]. Medications that affect neuropathic pain such as gabaminergic agents (e.g. gabapentin), or tricyclic antidepressants (e.g. imipramine, amitriptyline) should be considered in addition to systemic analgesics [26]. Pain management strategies for this group of patients are summarized in Table 2. In addition, dietary instruction, frequent use of saline/bicarbonate rinses, and good oral hygiene should be addressed. Finally, a number of medical devices typically described as mucosal coating agents (eg: Biotene ®, Gelclair®, Mugard ®, Gelclair ® and others), has been cleared by the FDA but data supporting impact on mucositis are limited and no guidelines have been developed by MASSC/ISOO to date. Nevertheless, these over-the-counter coating agents may be soothing and promote temporary oral comfort.

**Table 2:** Pain management Techniques in oncology (modified from Epstein et al.; Orofacial pain in Cancer; Part II- Clinical Perspective and Management; J Dent Res 86(6)2007

Palliative Radiation Therapy
Topical Analgesics/topical anesthetics
Systemic analgesics
Adjunctive medications (eg: Anxiolytics, Anticonvulsants, Antidepressant)
Physical and rehabilitative therapy: (massage/Physiotherapy; Cold/moist heat compresses)
Photobiomodulation/acupuncture
Psychologist support (Cognitive behavioral therapy, hypnosis)

In addition to mucositis, acute oral/dental infection during radiation therapy may be a cause for cancer treatment interruptions and excess morbidity. Extractions or other surgical procedures are not indicated during cytoreductive therapy. Hence, the alternative to surgical treatment is medical management, using antibiotics and analgesics that may control, but not resolve the infection, and create additional issues for the patient. Dental disease prevention must include regular daily fluoride applications, best applied via custom-made fluoride trays; when not able to be used (e.g. if severe mucositis develops), application by brushing on teeth or rinse application can be substituted. Fluoride application must be continued on a daily basis as long as dry mouth persists. In these patients, it is also important to provide a topical calcium source for the teeth in addition to fluoride. Special toothpastes (e.g. Enamelon ®) have been designed for this purpose.

Patient education is an integral part of dental treatment in order to support nutrition, reduce alcohol and tobacco use, and receive optimal oral care, which are important for both oral health and effective completion of cancer therapy [27, 28]. In addition, other factors such as taste changes, mucosal sensitivity, and xerostomia may negatively impact diet and nutritional intake, which may affect oral and general health. As a consequence, oral symptoms need attention from both oncologists and dental professionals in order to ensure timely diagnosis and appropriate treatment, as well as patient compliance with treatment recommendations.

## Post HNC treatment

Cytotoxic treatment-induced oral complications can be severe, and impact not only the quality of life, but also cancer therapy outcomes. This can be challenging, as patients are known to under-report oral complications to their medical providers during and following cancer therapy. HNC patients should be monitored closely to reinforce prevention, early diagnosis and management of late complications, and optimal oral care to prevent these complications. Timely identification of cancer recurrence and/or metastasis, leading to early referral is also indicated [29]. Effective management of oral infection, residual mucositis, sensory changes (mucosal pain, taste change/loss), reduced/altered salivation, dental and periodontal disease, soft tissue/bone necrosis, and temporomandibular joint disorders must be integral part of post-cancer therapy care [30]. Follow-up dental visits should be tailored and individualized based on patient conditions. At least twice per year check-ups are recommended, although an every 2–3 month schedule may be indicated for some cases (Table 1).

Patients should be instructed in daily atraumatic tooth brushing, bland oral rinses, flossing and fluoride gel applications, as well as management of mucositis and other residual sequelae, based on the MASCC/ISOO evidence-based guidelines [2, 11]. Ultrasonic or electric brushes may be recommended, although soft or supersoft manual toothbrushes are standard [31]. Prescription toothpaste/gel with 5000 ppm fluoride are recommended for dentate patients, but if the patient cannot tolerate due to mucositis, fluoride rinses may be substituted for short periods; return to brushing with prescription toothpaste should be conducted as soon as possible and maintained for the duration of the patient’s dentate life.

Patients skilled at dental flossing should be instructed to continue, avoiding trauma to the gingival tissue. Other interdental cleaning aids may be chosen if they are effective, and can be used atraumatically [32, 33]. Diet and supplements that are rich in carbohydrates, as well as sucrose-sweetened medications should be avoided, or when needed to support energy intake, should be taken with meals, and may be best after oral hygiene is performed [34].

## Management of specific oral complications related to HNC

### Mucositis

Mucositis is recognized as a critical, dose-limiting toxicity of current HNC cytotoxic therapy and its prevalence approaches 100 %. Acute signs and symptoms typically develop during second or third week of therapy and may continue for weeks to months following completion of treatment, particularly in those patients treated with chemoradiation [35]. Chronic mucosal sensitivity may be increased in those who experienced severe acute mucosal damage [33]. Radiation-induced loss of stem cells in the basal layer interferes with the replacement of cells in the superficial mucosal layers when they are lost through normal physiologic sloughing. The subsequent denuding of the epithelium results in mucositis, which can be painful and interfere with oral intake and nutrition. Chemotherapy can have a similar effect on the mucosa. Patient education includes frequent use of non-medicated bland oral rinses, possible use of film forming, lubricating mucosal coating agents, and topical analgesics/anesthetics as needed. Mouthwashes with alcohol, foods with acidic or spicy qualities, and coarse or abrasive foods should be avoided [36, 37]. Thorough and consistent oral hygiene is recommended.

Active research continues to evaluate novel prevention and treatment approaches to mucositis for future clinical application. Current management is focused on palliation of symptoms with topical and systemic analgesics. It is also important to recognize that pain due to mucositis is related to tissue damage and inflammation, resulting in nociceptive pain, but also due to neuropathic sensitization (neuropathic pain), which can be treated with neurologically active medications such as gabapentin and doxepin [37–41].

### Oral mucosal infections – viral, bacterial and fungal infections

Patient monitoring is critical in diagnosis of viral, bacterial and fungal infections. Diagnosis can be challenging during the period of mucositis, as there may be considerable overlap in signs and symptoms of mucositis and infection. Viral infections such as Herpes simplex virus (HSV), and other herpes viruses are common in patients treated with chemotherapy, while HSV reactivation is uncommon in HNC patients treated with radiation alone. Acyclovir and valcyclovir are considered equally effective in HSV prevention and treatment [42, 43]. Oral manifestations of viral infection may be more severe, with altered presentation and a more protracted course in cancer survivors. The more severe reactivation of Varicella-Zoster virus (shingles) requires larger doses of antiviral agents, which may be combined with systemic corticosteroids.

Bacterial infection occurs most commonly in association with dental or periodontal infection that may worsen during cancer treatment. For suitable selections of antibiotics (begin on an empiric basis for oral and dental infection), bacterial culture and antibacterial sensitivity tests may be required due to potential shifts in oral colonization, particularly in hospitalized patients, and in patients not responding to therapy as expected [34].

Oral yeast infection, most commonly due to *Candida albicans* is found in 7.5, 39.1 and 32.6 % prior to, during and post HNC therapy, respectively [44]. Clinical presentation of oropharyngeal candidiasis may be variable and not easily recognized, presenting as white patches, erythematous patches or plaques, as hyperplastic candidiasis or mixed presentation [44]. Recognition of infection may be challenging due to multiple presentations and overlap with other mucosal changes such as mucositis. Other symptoms such as coated sensation in the mouth, burning sensation and taste change can be associated with candidiasis and should raise attention.

Topical therapy for local disease is always preferable, but systemic antifungals may be necessary in the setting of immune deficiency or loss of mucosal integrity. Oncologists need to be aware that topical antifungal agents have had inconsistent efficacy and some are high in sugar, which reduces efficacy and increases dental caries risk in dry mouth dentate patients. Antifungals that are not sugar sweetened (e.g. vaginal suppositories), and oral systemic agents are more effective [45, 46]. Furthermore, additional fungal species with increased resistance to antifungal agents are observed in cancer patients, and therefore the principles of culture and identification of the fungal species may be important to guide therapy, particularly in non-responding cases [44].

### Other oral complications and their management

Other oral complications in HNC survivors are common. The key is to identify risk factors and early onset symptoms, and to offer referral for expert care. For example, HNC patients receiving cytotoxic chemotherapy and radiotherapy can have dry mouth or xerostomia that continues indefinitely due to reduced saliva production (hyposalivation) [47, 48]. Dry mouth is a common and significant chronic complication and drives late oral complications; some patients may accommodate to ongoing hyposalivation over time and may report reduced or even no symptoms. However, effects of reduced saliva on the health of oral tissues continue. Hyposalivation management includes assessment of saliva production, with first approach to treatment being stimulation of any residual function (chewing and taste stimulation, systemic sialagogues such as pilocarpine, civemiline, or bethanechol), and palliative topical products [49]. In addition to reduced production of saliva, changes in the quality of saliva, with thickened or viscous secretions can be challenging to manage, and trials of mucolytic agents may be considered (guaifenesin, acetyl-cysteine). Encouraging good daily oral hygiene, frequent dental visits, and dental fluoride and remineralization supplementation can help counteract the changes induced by insufficient salivation. For those patients with persisting hyposalivation, wetting of oral surfaces, and replacement of calcium, phosphate, and use of antimicrobial rinses have been shown to be safe and effective in relieving symptoms of dry mouth and minimizing infection potential. Dry mouth-associated taste change, gastrointestinal upset, mucosal sensitivity, inflammation and ulceration [50] significantly limit food intake, and may lead to dietary compromise that complicates nutrition and general health. Dry mouth increases risk of dental demineralization and tooth cavitation that can lead to rampant dental damage, increase risk for progression of periodontal disease and increase risk of osteoradionecrosis of the jaw (ORN). Dental sensitivity to temperature is also associated with lower saliva secretion and lower salivary pH. Oral burning sensation due to mucosal sensitivity is associated with hyposalivation and arises from peripheral neuropathy [2, 51]. In addition, 50–75 % of HNC patients have dysgeusia, which contributes to the reduction in quality of life. Radiation- and chemotherapy-induced neuropathies are associated with inflammation, neurotoxicity, oxidative stress, and ischemia, which may persist long after clinical mucositis resolved [52, 53].

Among other complications, ORN is a known risk following head and neck radiotherapy. It is associated with dental disease and dental procedures, but can occur spontaneously, without identified stimulus. This risk may be increased in people on medications such as bisphosphonates or denosumab for osteoporosis, people with diabetes, on immunosuppressives, and in tobacco users [54]. Prevention of ORN is the key concept and it reinforces the need for dental stabilization before cancer therapy, and continuing good oral/dental health in survivors. Management of ORN after onset must be individualized in coordinated dental and medical care.

Current treatment strategies are based on limited research and clinical anecdotes and thus, should be undertaken by experienced healthcare providers due to the complexity of care. Hyperbaric oxygen (HBO) therapy and use of antiseptic oral rinses, and sometimes antibiotics when secondary infection is diagnosed, has been the medical approach to therapy [55]. However, the use of HBO is expensive and controversial and no good evidence for or against it has been published. More recently medical management with local infection management and pentoxifylline and Vitamin E has been supported in phase II clinical trials [51]. If necrosis develops, continuation of these medications is recommended with the addition of clodronate to stimulate new bone formation [56]. As necrotic bone cannot be regained, the goal of treatment is prevention of progression, pain control, and management of secondary infection in hard and soft tissues (osteomyelitis, cellulitis). Surgical management with local sequestrectomy or vascularized tissue transfer may be needed [57].

Post treatment fibrosis is best managed by early recognition and physical therapy. Pentoxifylline and vitamin E has been suggested [58].

Recurrent or second primary oral malignancy is a known risk and people with prior upper aerodigestive tract cancer are the highest risk population for a post-treatment malignant lesion. Post-radiation mucosal changes can make detection of early changes difficult, and the potentially delayed healing in the high dose radiation fields make determination to biopsy more challenging.

Speech and swallow therapists and nutrition/dietary instruction are important aspects of care in the HNC patient, and these professionals are important part of the multidisciplinary HNC team.

## Conclusion

The number of HNC survivors has increased, due mostly to change in risk factors for cancer (HPV), and advances in cancer therapeutics and supportive care. This growing population requires specialized oral care by personnel trained in managing the intricacies of these complex patients. Oncologists and dentists must collaborate to optimize care, as well as to increase knowledge of preventive and therapeutic options for oral health maintenance, oral cancer detection and suitable referral conditions with pathways. The oncology team should include dental professionals specialized in head and neck cancer survivor management, which will contribute to better oral complication prevention, detection, and treatment, and an improved quality of life.

## Abbreviations

HNC: Head and neck cancer
HPV: Human papilloma virus
HSV: Herpes simplex virus
LLLT: Low-energy laser
MASCC/ISOO: Multinational Association of Supportive Care in Cancer/International Society of Oral Oncology
mTOR: Mammalian target of rapamycin
ONJ: Osteoradionecrosis of the jaw
